# Factual survey of the clinical use of deformable image registration software for radiotherapy in Japan

**DOI:** 10.1093/jrr/rrz034

**Published:** 2019-05-24

**Authors:** Noriyuki Kadoya, Satoshi Kito, Masahiko Kurooka, Masahide Saito, Akihiro Takemura, Naoki Tohyama, Masahide Tominaga, Yujiro Nakajima, Yukio Fujita, Yuki Miyabe

**Affiliations:** 1 Department of Radiation Oncology, Tohoku University Graduate School of Medicine, Sendai, Japan; 2 Department of Radiotherapy, Tokyo Metropolitan Cancer and Infectious Diseases Center Komagome Hospital, Tokyo, Japan; 3 Department of Radiology, Tokyo Medical University, Tokyo, Japan; 4 Department of Radiology, University of Yamanashi, Yamanashi, Japan; 5 Faculty of Health Sciences, Institute of Medical, Pharmaceutical and Health Sciences, Kanazawa University, Kanazawa, Japan; 6 Department of Radiation Oncology, Tokyo Bay Advanced Imaging and Radiation Oncology Clinic Makuhari, Chiba, Japan; 7 Department of Therapeutic Radiology, Institute of Biomedical Sciences, Tokushima University Graduate School, Tokushima, Japan; 8 Department of Radiological Sciences, Faculty of Health Sciences, Komazawa University, Tokyo, Japan; 9 Department of Radiation Oncology and Image-applied Therapy, Kyoto University Graduate School of Medicine, Kyoto, Japan

**Keywords:** deformable image registration, radiotherapy, segmentation, propagation, dose accumulation

## Abstract

Deformable image registration (DIR) has recently become commercially available in the field of radiotherapy. However, there was no detailed information regarding the use of DIR software at each medical institution. Thus, in this study, we surveyed the status of the clinical use of DIR software for radiotherapy in Japan. The Japan Society of Medical Physics and the Japanese Society for Radiation Oncology mailing lists were used to announce this survey. The questionnaire was created by investigators working under the research grant of the Japanese Society for Radiation Oncology (2017–2018) and intended for the collection of information regarding the use of DIR in radiotherapy. The survey was completed by 161 institutions in Japan. The survey results showed that dose accumulation was the most frequent purpose for which DIR was used in clinical practice (73%). Various commissioning methods were performed, although they were not standardized. Qualitative evaluation with actual patient images was the most commonly used method (28%), although 30% of the total number of responses (42% of institutions) reported that they do not perform commissioning. We surveyed the current status of clinical use of DIR software for radiotherapy in Japan for the first time. Our results indicated that a certain number of institutions used DIR software for clinical practice, and various commissioning methods were performed, although they were not standardized. Taken together, these findings highlight the need for a technically unified approach for commissioning and quality assurance for the use of DIR software in Japan.

## INTRODUCTION

Deformable image registration (DIR) has recently become commercially available in the field of radiotherapy [1–3]. This is an exciting and interesting technology for multi-modality image fusion, anatomic image segmentation, four-dimensional (4D) dose accumulation and lung function imaging [4–9]. Many studies have shown the potential of DIR-based technologies for improving treatment outcomes and quality of radiotherapy [1–6]; for example, Yeo *et al.* demonstrated that DIR-based dose-warping can yield accurate predictions of dose distribution for a range of mass- and density-conserving deformations representative of those observable in anatomical targets [6].

Several guidelines for DIR in clinical radiotherapy have recently been published, including task group 132 (TG 132) from the American Association of Physicists in Medicine (AAPM) and DIR Guideline 2018 from the Japanese Society for Radiation Oncology [7, 8]. According to these guidelines, the number of institutions using DIR software in clinical practice is expected to increase annually. However, there is no quality assurance program to establish the safe clinical use of DIR software in Japan.

Through better understanding of the current status of clinical use of DIR software for radiotherapy in Japan, we can consider the next steps for developing a quality assurance program and promoting the widespread use of the software in Japan. Here we present a survey of the current status of the clinical use of DIR software for radiotherapy in Japan.

## MATERIALS AND METHODS

The survey was announced in January 2018 on the mailing lists of the Japan Society of Medical Physics and the Japanese Society for Radiation Oncology. The questionnaire was created by the investigators working under the research grant of the Japanese Society for Radiation Oncology and intended for the collection of information regarding DIR use in clinical radiotherapy.

The survey questions included the following: (i) the possession rate; (ii) clinical purposes; (iii) parameter settings; (iv) operators (occupations); (v) subject treatment site; (vi) type of contour for segmentation; (vii) modification rate of autosegmented contour by a manual delineation; (viii) treatment site where DIR software cannot work well; (ix) types of image modality for DIR; (x) methods of commissioning; and (xi) whether you want to use the DIR software (only asked at the institutions without DIR software).

It should be noted that radiotherapy treatment planning systems, such as Eclipse (Varian Medical Systems, Palo Alto, CA, USA) and RayStation (RaySearch Laboratories, Stockholm, Sweden), were considered as the DIR software packages, when these systems had DIR-related modules.

## RESULTS

**Fig. 1.**
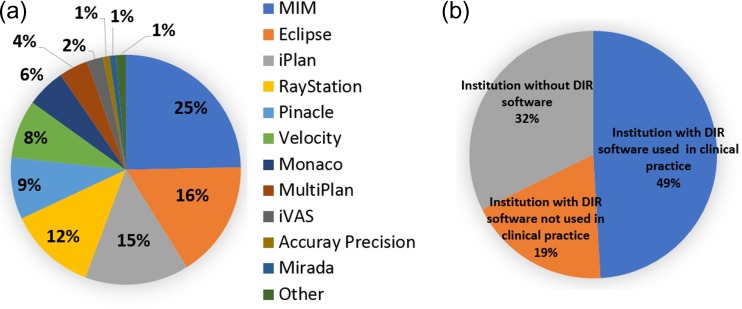
Pie charts showing the type of DIR software used (**a**) and the percentage of institutions using DIR software (**b**).

The survey was completed by 161 institutions in Japan. Figure 1 presents the results for the type of DIR software used and the percentage of institutions that use the DIR software and those that do not. MIM software (MIM Software Inc., Cleveland, OH, USA) was most frequently used in clinical practice (25%) followed by Eclipse software. The number of institutions without DIR software, with DIR software used in clinical practice and with DIR software not used in clinical practice were 52 (32%), 79 (49%) and 30 (19%), respectively. Thus, ~50% of institutions used DIR software in clinical practice.

**Fig. 2.**
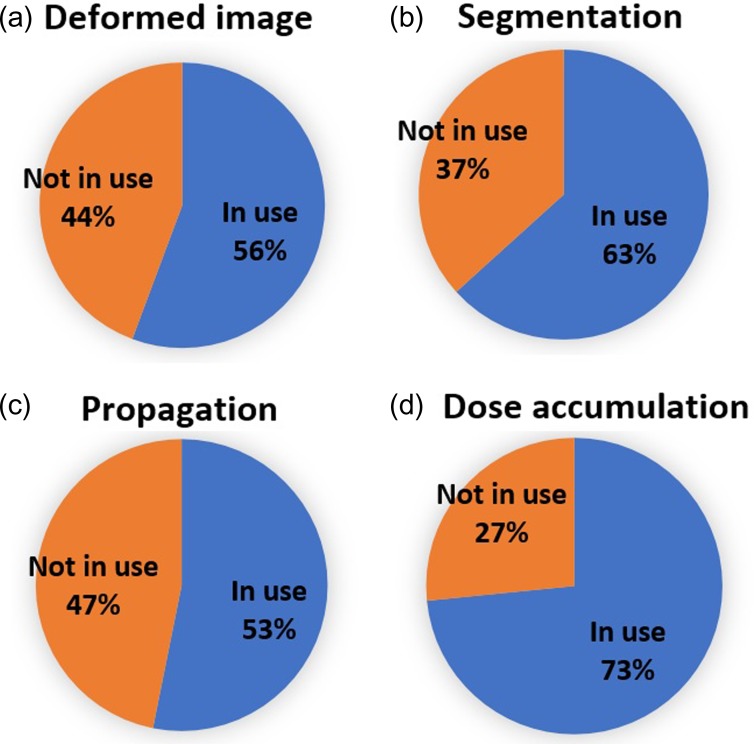
Pie charts showing the ratio of clinical use of DIR software based on purpose.

**Fig. 3.**
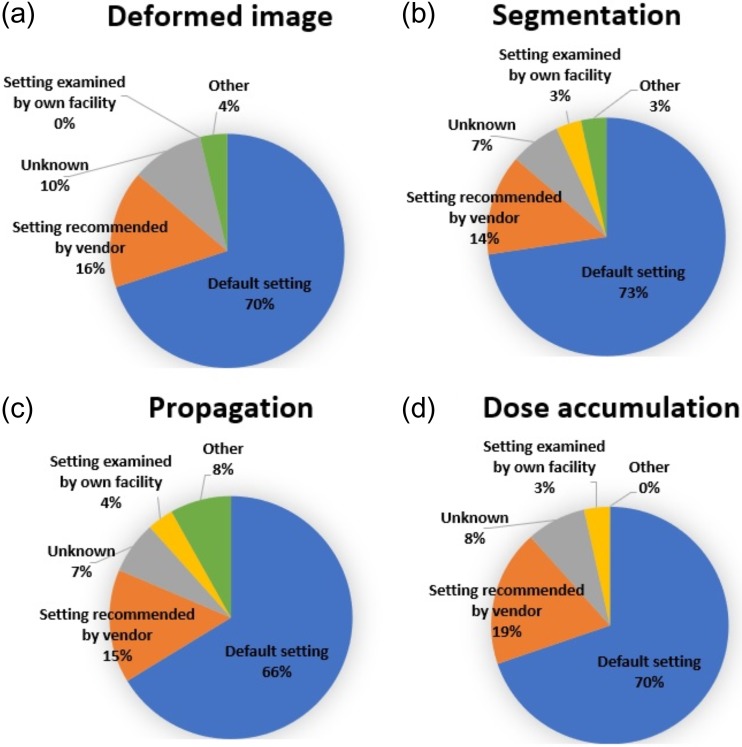
Pie charts showing the DIR parameters used in clinical practice.

Figure 2 presents the ratio of clinical use of DIR software according to the purposes, such as for deformed image and segmentation. The ratios of the clinical use for deformed images, segmentations, propagations and dose accumulations were 56, 63, 53 and 73%, respectively. This indicates that dose accumulation was the most frequent purpose for which DIR was used in clinical practice. Figure 3 shows information regarding the DIR parameter settings used in clinical practice. For all intended purposes, the majority of institutions employed the default parameter settings of DIR (e.g. dose accumulation: 70%). The second most common parameter setting was that recommended by the vendor (e.g. dose accumulation: 19%).

**Fig. 4.**
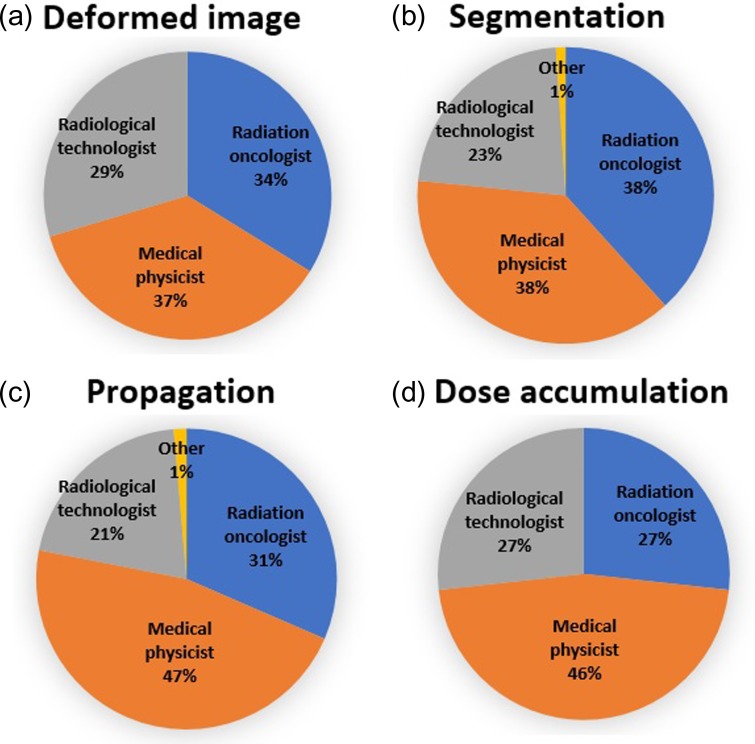
Pie charts showing the occupations of DIR software operators.

**Fig. 5.**
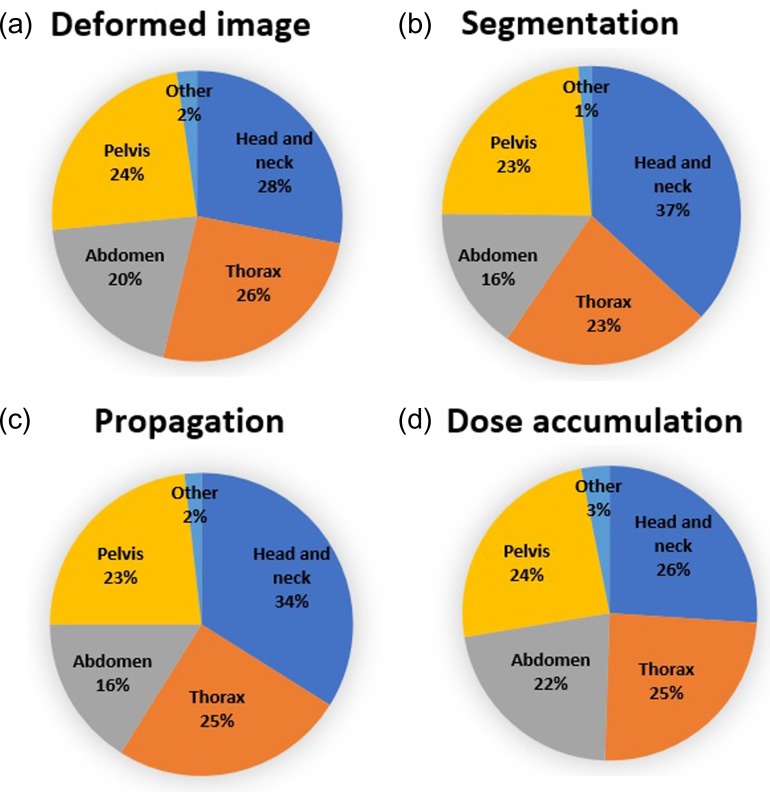
Pie charts showing the subject treatment site for each intended use of DIR.

Figure 4 presents the data regarding the occupation of the operators of DIR software. Operators were most commonly medical physicists, particularly for the purposes of propagation and dose accumulation. Data regarding subject treatment sites for each intended use of DIR are shown in Fig. 5. The first and second most common sites were head and neck and thoracic sites, respectively.

**Fig. 6.**
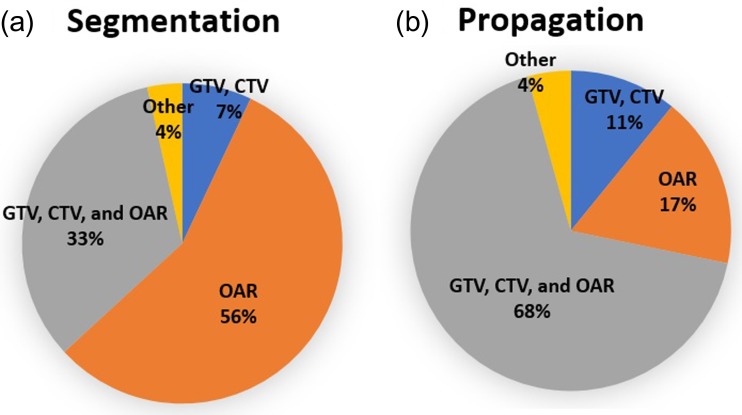
Pie charts showing the type of contour for segmentation.

**Fig. 7.**
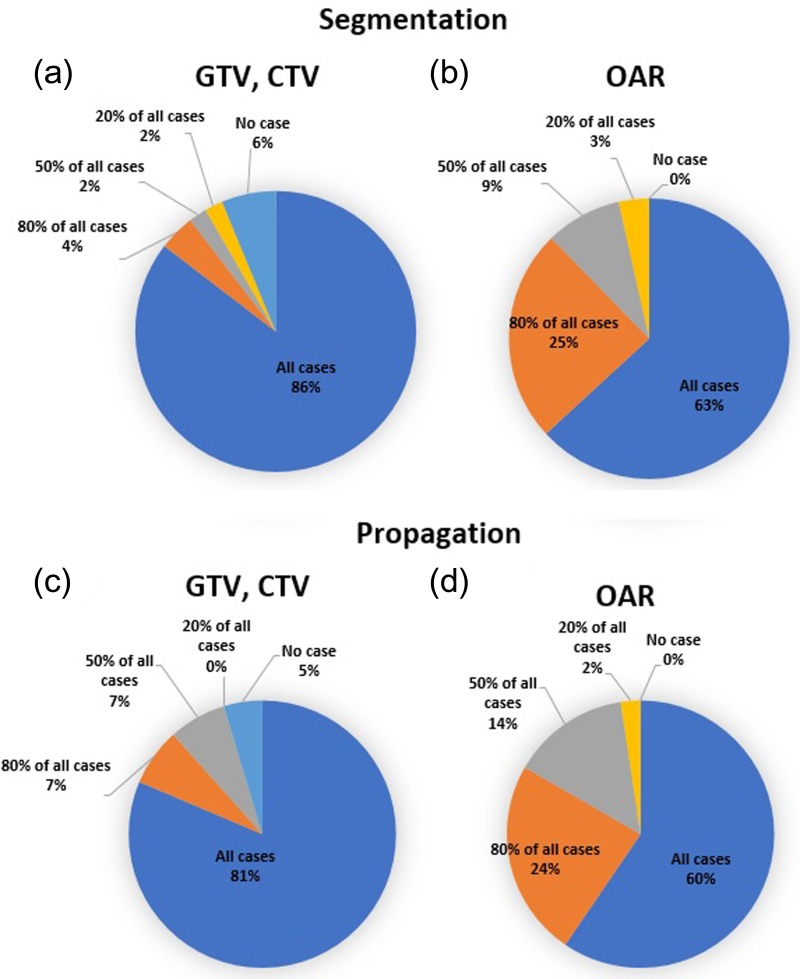
Pie charts showing the ratio of modification of contour for segmentation and propagation.

**Fig. 8.**
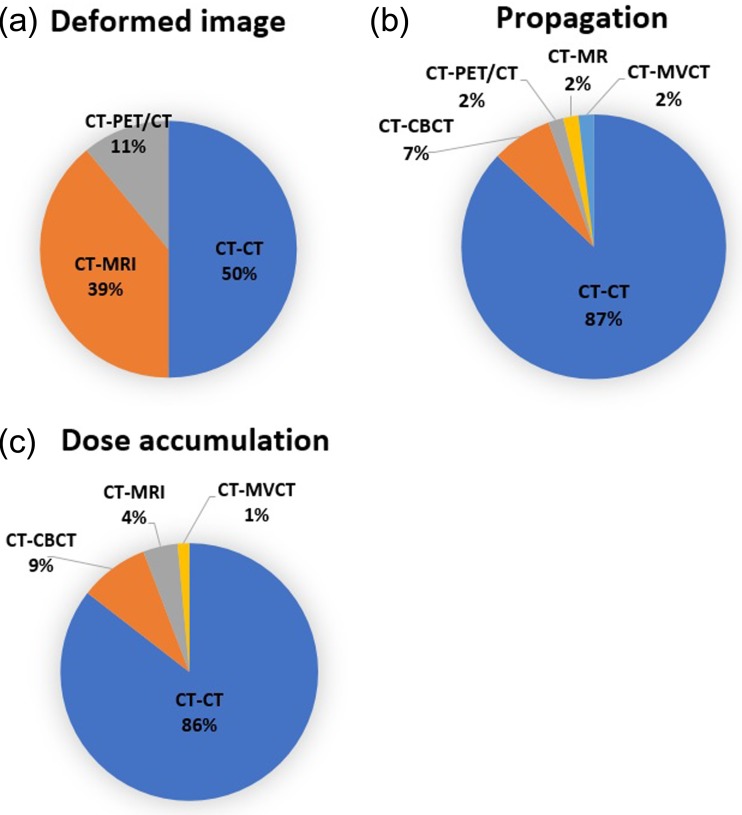
Pie charts showing the combination of image modalities for DIR.

**Fig. 9.**
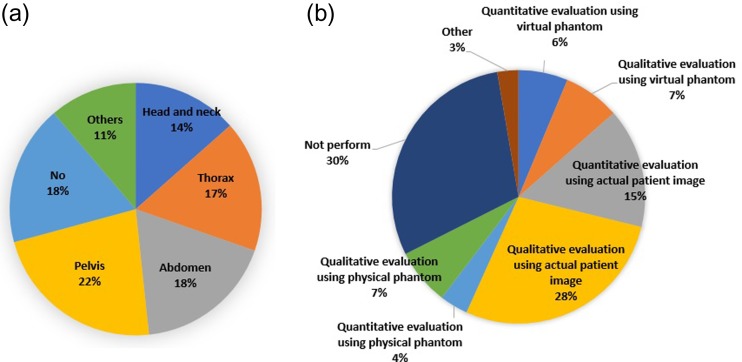
Pie charts showing the ratio of treatment sites with a risk of deformation (**a**) and method of commissioning (**b**).

Figure 6 shows the findings for the type of contour for segmentation. For segmentation, the majority of institutions used segmentation only for organ at risk (OAR) delineation (56%) and for propagation; the majority of institutions used segmentation for gross tumor target volume (GTV), clinical target volume (CTV) and OAR (68%). For both segmentation and propagation, the majority of institutions modified the contours of both GTV/CTV and OAR for all cases (Fig. 7). DIR between computed tomography (CT) and CT images was used most frequently; however, that between CT and magnetic resonance images was common for the clinical use of deformed images (Fig. 8). The pelvis was reported to be the most challenging region (Fig. 9a).

With respect to commissioning, various methods were performed, although they were not standardized (Fig. 9b). Qualitative evaluation with actual patient images by a visual inspection was the most commonly used method (28%). However, 30% of the total number of responses (42% of institutions) indicated not performing commissioning; this question allowed multiple choices.

## DISCUSSION

In this study, we surveyed the current status of the clinical use of DIR software for radiotherapy in Japan. We found that 49% of institutions surveyed owned DIR software and used it in clinical practice. Among these institutions, dose accumulation was the most common clinical purpose for which DIR was used (73%). We found that various methods were used for commissioning, although they were not standardized. Qualitative evaluation using actual patient images was the most common method (28%), although 30% of the total number of responses (42% of institutions) indicated not performing commissioning.

In terms of clinical purpose, DIR was most frequently used for dose accumulation. DIR-based dose accumulation is an attractive tool for radiotherapy because it can accurately calculate accumulated doses that cannot be calculated by conventional methods, such as simple dose–volume histogram (DVH) parameter addition and rigid registration-based dose accumulation methods. For example, Andersen *et al.* and Kadoya *et al.* [9, 10] reported a dosimetric difference in accumulated external beam radiotherapy and intracavitary brachytherapy doses between simple DVH parameter addition and DIR-based dose accumulation. When we add the two dose distributions with different dose per fraction (e.g. 50 Gy/25 fr and 24 Gy/4 fr), we may need to consider the biological effects of different fraction doses in the dose summation process. For example, MIM software calculates a biologically effective dose (BED) using several common dose–response models based on the linear–quadratic formalism. Calculating the BED allows a physical dose to be converted into a dose that describes the biological effect of the radiation on tumor and normal tissue. It should be noted that some commercially available DIR software cannot consider the biological effect on the dose summation process.

The second most common reported use of DIR was for propagation. The average physician working time for the design of the respective head and neck treatment contours was 2.7 h for IMRT compared with 0.3 h for conventional 3D radiotherapy [11]. Thus, DIR-based auto-propagation can greatly reduce contouring time. Several papers have been published on auto-propagation accuracy [12–14]. Loi *et al.* evaluated DIR accuracy using synthetic images generated with the ImSimQA (Oncology Systems Limited, Shrewsbury, UK) by applying a specific deformation vector field to real patient data sets from 13 institutions using six commercial DIR software packages [14]. They reported that subvoxel accuracy was achieved in the head and neck for all algorithms, although large errors were observed in low-contrast regions that underwent significant deformation, such as the pelvis. Their finding that the pelvis was the most challenging site for most algorithms is consistent with our survey results (Fig. 9a). Therefore, the operator of the DIR software should pay attention to DIR accuracy, especially for the pelvic region.

Next, in terms of commissioning of the DIR software, various methods have been performed, indicating that the methods are not standardized. Qualitative evaluation using actual patient images was the most commonly used method (28%). However, 42% of institutions did not perform commissioning. DIR accuracy is reported to depend strongly on both the DIR software and procedure (e.g. DIR parameter settings) [4, 15, 16]. Kadoya *et al.* evaluated the commercially available DIR software using thoracic 4D CT images from multiple centers, and found that DIR accuracy differed among institutions because it was dependent on the DIR software and procedure. Thus, commissioning of the DIR software is important for understanding the basis of registration and determining the optimal DIR parameters for each treatment site. According to TG 132 by the AAPM, physical and digital phantoms are useful tools for the commissioning of DIR. In the dosimetric guidelines, there are no specific recommendations for commissioning of DIR, although overviews of qualitative and quantitative evaluation methods were introduced. Qualitative evaluation using actual patient images, which was the most frequently reported method in our survey, is a simple method, although there can be large variance in assessment results among evaluators. Thus, quantitative evaluation is preferred to qualitative methods. The AAPM has provided several digital phantoms to be used for quantitative evaluation for commissioning. Given the widespread use of DIR software in Japan, a similar system should be established. In addition, a physical phantom is also useful to do the commissioning and quality assurance of the DIR software because this phantom can be used for the end-to-end quality assurance test, which ensures accurate data representation, image transfer and integrity verification between image acquisition devices, image registration systems and other radiotherapy systems that use the image registration results [8]. A physical phantom has the great potential for evaluating the accuracy of DIR-based dose accumulation directly [17–21]. We recommend that comprehensive commissioning and quality assurance should use these three data: physical phantom, digital phantom and clinical patient data due to different advantages and disadvantages of each method. Ideally, it is desirable to compare DIR results obtained by these methods in each institution with the results in other institutions for validation of the obtained DIR result. There are few commercially available physical phantoms suitable for the evaluation of DIR accuracy. Development of a physical phantom is still ongoing.

## CONCLUSIONS

We surveyed the current clinical use of DIR software for radiotherapy in Japan. Our results revealed that many institutions use a DIR software package in clinical practice. Among these institutions, dose accumulation was the most common reason for DIR use (73%). Various commissioning methods were performed, although they were not standardized. Qualitative evaluation using actual patient images was the most commonly used method (28%), although 30% of the total number of responses (42% of institutions) reported that they did not perform commissioning. Taken together, these findings highlight the need for a technically unified approach for commissioning and quality assurance for the use of DIR software in Japan.
